# (S)-crizotinib reduces gastric cancer growth through oxidative DNA damage and triggers pro-survival akt signal

**DOI:** 10.1038/s41419-018-0667-x

**Published:** 2018-05-31

**Authors:** Jiansong Ji, Weiqian Chen, Weishuai Lian, Ruijie Chen, Jinqing Yang, Qianqian Zhang, Qiaoyou Weng, Zia Khan, Jie Hu, Xi Chen, Peng Zou, Xiaoming Chen, Guang Liang

**Affiliations:** 1 Chemical Biology Research Center, School of Pharmaceutical Sciences, Wenzhou Medical University, Wenzhou, Zhejiang 325035 China; 2 Department of Interventional Radiology, The Fifth Affiliated Hospital of Wenzhou Medical University, Lishui, Zhejiang 323000 China; 3 Department of Interventional and Vascular Surgery, Shanghai Tenth People’s Hospital, Tongji University, Shanghai, 200072 China; 4 Department of Pharmacy, The Second Affiliated Hospital of Wenzhou Medical University, Wenzhou, Zhejiang 325000 China; 5 The First Affiliated Hospital of Wenzhou Medical University, Wenzhou, Zhejiang 325035 China

## Abstract

Gastric cancer (GC), a common gastrointestinal malignancy worldwide, has poor prognosis and frequent recurrence. There is a great need to identify effective therapy for GC. Crizotinib is a multi-targeted, clinically available oral tyrosine kinase inhibitor approved for lung cancer, but its use for the highly heterogeneous disease of GC is unknown. The goal of this study was to investigate the anti-cancer mechanisms of the (S)-crizotinib in inhibiting GC growth. Human GC cell lines (SGC-7901 and BGC-823) and the (S)-crizotinib-resistant BGC-823/R were cultured for determining the effects of (S)-crizotinib on cell viability, apoptosis, oxidant generation, and cell cycle progression. Involvement of ROS, Akt signaling, MTH1, and DNA damage was tested with respective pharmacological blockade. The in vivo anti-tumor effects of (S)-crizotinib were determined using xenograft tumor mice. Results indicated that (S)-crizotinib decreased GC cell viability, induced growth arrest and apoptosis, and increased levels of γH2AX and Ser1981-phosphorylated ATM, which were inhibited by NAC. The anti-cancer mechanism of (S)-crizotinib was independent of MTH1. Moreover, ATM-activated Akt, a pro-survival signal, whose inhibition further enhanced (S)-crizotinib-induced inhibition of GC cell growth and tumor growth in xenograft mice, and re-sensitized resistant GC cells to (S)-crizotinib. (S)-crizotinib reduced GC cell and tumor growth through oxidative DNA damage mechanism and triggered pro-survival Akt signaling. We conclude that inclusion of Akt inhibition (to block the survival signaling) with (S)-crizotinib may provide an effective and novel combination therapy for GC in the clinical setting.

## Introduction

Gastric cancer (GC), a common malignancy worldwide, is the second leading cause of cancer-related deaths globally and the third leading cause in developed countries^1,2^. Despite advances in management of GC patients with distant metastasis, high recurrences and poor prognosis remain, with limited treatment options and a median survival of <1 year^3,4^. An added challenge is that GC is a highly heterogeneous disease, its etiology multifactorial, with complex host genetic and environmental factors contributing to its development^3–6^. To-date, only a handful of targeted molecular therapeutic agents, e.g., trastuzumab (anti-epidermal growth factor receptor 2 (ERBB2) antibody) and ramucirumab (anti-VEGFR2 antibody), have been approved by the US Food and Drug Administration for those patients identified with the respective genetic defects^3–5,7^, but the majority of GC patients must still rely on the current standard of care with chemotherapy and/or surgical resection^3–5,7^. Thus, there is an urgent need to better understand the pathogenesis of GC and to identify more effective, less toxic therapeutic strategies.

A recent genomic profiling study by Ali et al.^5^ indicated 1 in 5 GC patient cases have clinically relevant alterations in RTKs. For management of advanced lung adenocarcinoma, there are clinically available, well-tolerated oral tyrosine kinase inhibitors (TKIs)^8^. In particular, crizotinib, an ATP-competitive, small-molecule multi-targeted TKI, exerts in vivo anti-tumor activity and in vitro activity against the kinase domains of RTKs, specifically, ALK (anaplastic lymphoma kinase), MET (hepatocyte growth factor receptor), and ROS1 (proto-oncogene receptor tyrosine kinase 1)^9^. These developments have led to a recent interest to evaluate therapeutic potentials of crizotinib for the highly heterogeneous disease of GC. To-date, only a handful of GC patients has been studied for crizotinib treatment, with inconclusive outcomes^3–5^. Limited preclinical studies reported that (S)-crizotinib, and not the (R)-enantimer, induces strong anti-proliferative effects of a panel of human cancer cell lines and inhibits xenograph tumor growth of SW480 cells^10^, which is believed to be attributed to inhibition of MTH1 (MutT Homolog 1), a nucleotide pool sanitizing enzyme^10,11^. These reports suggest that (S)-crizotinib, clinically available with minimal toxicity, could be a potentially important therapy for GC patients.

The goal of this study was to investigate the anti-cancer mechanisms of (S)-crizotinib in inhibiting GC growth. Our results indicated that (S)-crizotinib’s anti-cancer activity in GC was through an oxidative DNA damage mechanism independent of MTH1. Moreover, (S)-crizotinib triggered pro-survival Akt signaling, suggesting that inclusion of Akt inhibition (to block pro-survival signaling) as part of (S)-crizotinib treatment strategy may provide an effective and novel combination therapy for GC in the clinical setting.

## Results

### (S)-crizotinib inhibits gastric cancer cell growth

**Fig. 1:**
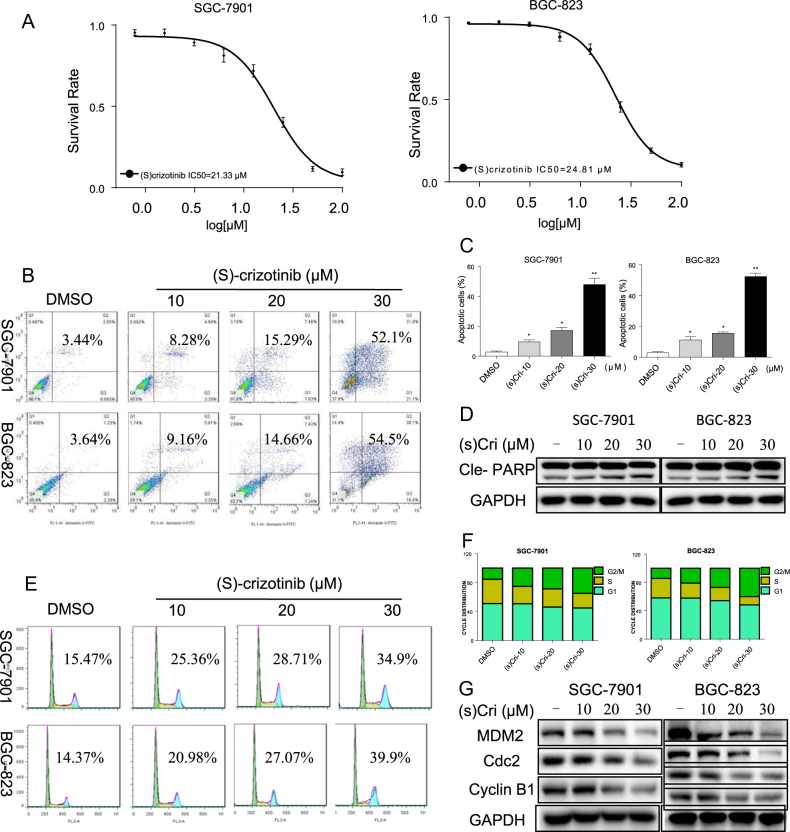
(S)-crizotinib inhibits gastric cancer cell growth. Cultured SGC-7901 and BGC-823 cells were exposed to increasing concentrations of (S)-crizotinib for 24 h, and in vitro cell growth assays prepared for the analysis as described below. **a** Viability of gastric cancer cells in response to (S)-crizotinib was measured by the MTT assay; average absorbance ± SEM values are graphed against (S)-crizotinib concentrations; *n* = 4; IC_50_ is indicated on lower left of graph. **b** Induction of apoptosis in response to (S)-crizotinib: was determined by annexin V/PI staining. DMSO was used as control; shown is representative flow cytometric analysis. **c** Quantification of apoptotic cells presented as percent total, average ± SEM; *n* = 4; (*p < 0.05, **p < 0.01 compared to DMSO). **d** Representative Western blot analysis of apoptosis-related, cle-PARP (Poly ADP-ribose polymerase) following exposure to (S)-crizotinib for 20 h; GAPDH was used as loading control; *n* = 4. **e** Induction of cell cycle arrest in gastric cancer cells was determined by PI staining following exposure to (S)-crizotinib for 14 h; shown is representative flow cytometric analysis. **f** Histograms showing cell cycle distributions from flow data collected from **e**; *n* = 4. **g** Representative Western blot analysis of key G2/M cell cycle-related proteins: murine double minute 2 (MDM-2), Cyclin B1, and cell division cycle protein 2 (Cdc2) in cells treated with (S)-crizotinib for 14 h; GAPDH as loading control; *n* = 4

The anti-cancer activity of (S)-crizotinib was investigated using two human GC cell lines, SGC-7901 and BGC-823, in which the RTKs have been reported to be highly activated.^12,13^ (S)-crizotinib decreased viability of both cell lines at comparable levels (IC_50 = _21.33 and 24.81 μM, respectively) (Fig. 1a), a finding consistent with cell rounding and decreased cell density (Figure S1). The effects of (S)-crizotinib on apoptosis of the GC cells were determined with annexin V/PI staining and detection by flow cytometry. (S)-crizotinib treatment increased the % apoptotic cells in a dose-dependent manner (Fig. 1b, c), and increased levels of Cle-PARP (Fig. 1d and S2). PARP is a well-characterized caspase substrate, and its cleaved products considered an indicator of apoptosis^14^. In addition, flow cytometric analysis of cell cycle progression of the GC cells revealed that (S)-crizotinib increased the proportion of cells in the G2/M phase, with a corresponding decrease in S phase, indicating increased number of cells at cell cycle arrest (Fig. 1e, f). Western blot analysis of (S)-crizotinib-treated cells indicated decreased expression of key regulators of the cell cycle: MDM2 protein 2 (regulator of p53)^15^, CDC2 (cell division cycle protein 2)^16^, and cyclin B 1^16^ (Fig. 1g).

### (S)-crizotinib induces ROS-dependent apoptosis and DNA damage

#### Intracellular oxidant production

**Fig. 2:**
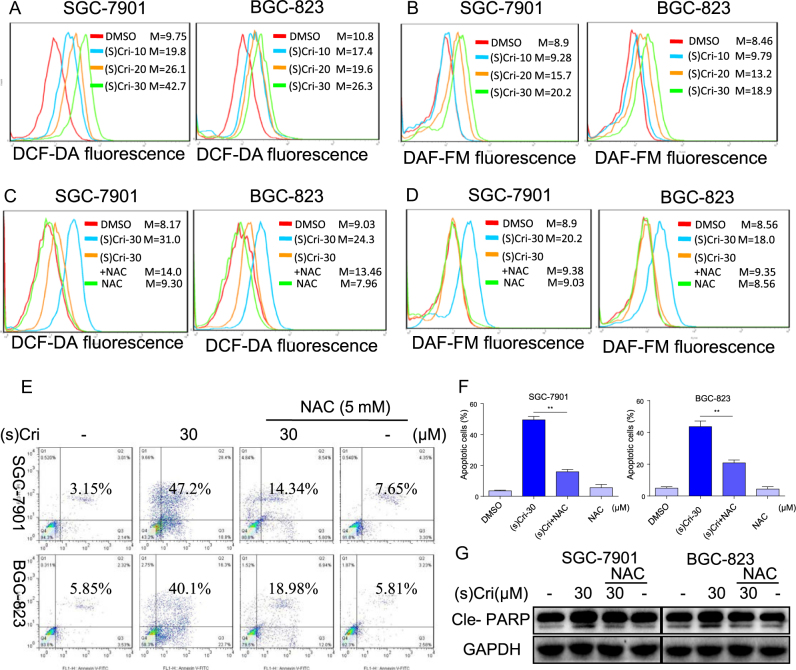
(S)-crizotinib induces oxidative stress and ROS-dependent apoptosis in human gastric cancer cells. SGC-7901 and BGC-823 cells were treated at indicated concentrations of (S)-crizotinib for 2 h (except where indicated) and prepared for analyses described as follows. **a** Intracellular ROS and **b** nitric oxide generation induced by (S)-crizotinib was measured using respective fluorescent dyes, DCFH-DA and DAF-FM-DA; shown are representative flow cytometric analyses; *m* = mean fluorescence intensity value; *n* = 4. The effects of NAC on (S)-crizotinib-induced oxidant generation in SGC-7901 and BGC-823 were determined by pretreatment with 5 mM N-acetyl cysteine (NAC) for 2 h prior to (S)-crizotinib (30 μM) treatment, and **c** ROS and **d** nitric oxide determined with DCFH-DA and DAF-FM-DA, respectively; *n* = 4. **e** Effects of NAC pretreatment (5 mM for 2 h) on (S)-crizotinib-induced apoptosis in 24 h; shown is representative flow analysis of the effects of NAC on cell apoptosis assayed by annexin V/PI staining. **f** Extent of apoptosis is determined by quantification of annexin V/PI stained cells; shown is average + SEM; *n* = 4; **p < 0.01. **g** Representative Western blot analysis of cle-PARP in cells pretreated with NAC prior to (S)-crizotinib treatment; *n* = 4

Evidence indicates that Crizotinib can induce oxidative stress in several cell types, i.e., ovarian cancer cells^17^, alveolar rhabdomyosarcoma cells^18^, and cardiomyocytes^19^. We determined the intracellular levels of ROS and nitric oxide (NO) in SGC-7901 and BGC-823 cells in response to (S)-crizotinib. The cells were treated with (S)-crizotinib for 2 h, and ROS production was detected with the fluorescent dye, 2’,7’-dichlorofluorescein diacetate (DCFH-DA) or NO generation with 4-amino-5-methylamino-2’,7’-difluorofluorescein diacetate (DAF-DA). Results indicated that (S)-crizotinib-induced dose-dependent increases in ROS (Fig. 2a) and NO (Fig. 2b).

#### ROS-induced apoptosis

We evaluated the relation of oxidative stress with (S)-crizotinib-induced apoptosis by using NAC to dampened intracellular ROS. NAC, a precursor of glutathione, interacts directly with ROS species as a scavenger^20^. The GC cells were pretreated with NAC (5 mM for 2 h) prior to (S)-crizotinib treatment. Results indicated that NAC pretreatment prevented the (S)-crizotinib-induced increases in ROS (Fig. 2c and S3) and NO level (Fig. 2d). NAC pretreatment reduced the (S)-crizotinib-induced increases in apoptotic cells by 60–70% as detected by annexin V/PI staining (Fig. 2e, f), which is consistent with decreased amounts of cle-PARP (Fig. 2g and S4).

#### Oxidative DNA damage

**Fig. 3:**
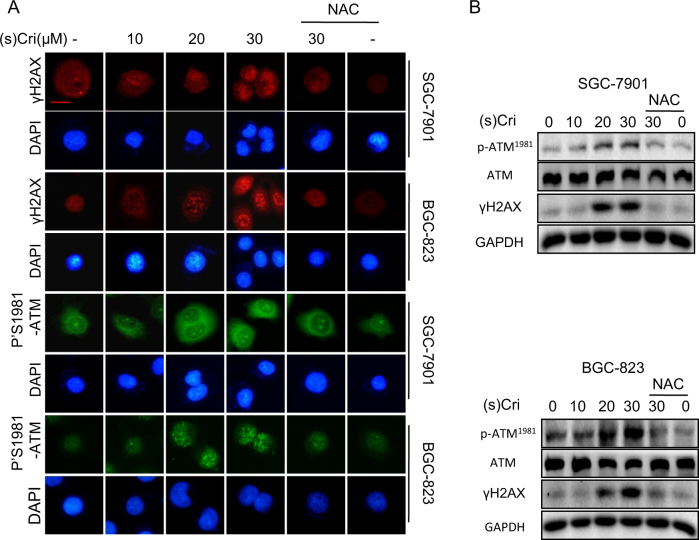
(S)-crizotinib induces oxidative DNA damage in gastric cancer cells. SGC-7901 and BGC-823 cells were treated with different concentrations of (S)-crizotinib for 10 h, while some groups were pretreated with NAC (5 mM for 2 h) prior to (S)-crizotinib, and assayed as follows. **a** DNA damage was determined by immunofluorescent detection of γH2AX and Ser-1981 phosphorylated ATM, nuclei were counterstained with DAPI; shown is representative fluorescent image; *n* = 4; scale bar = 10 μm. **b** Representative Western blot analysis of Ser-1981-phosphorylated ATM (pATM^1981^), γH2AX, and ATM from the treatment groups; GAPDH as loading control, *n* = 4

We suspected that (S)-crizotinib-induced oxidative stress likely causes DNA damage in the GC cells, i.e., base modifications, double-strand breaks. The levels of phosphorylated histone 2AX (γH2AX)^21,22^ and ATM^23,24^ were determined as indices of DNA damage in SGC-7901 and BGC-823 cells treated with (S)-crizotinib. Results indicated that (S)-crizotinib increased levels of γH2AX and Ser1981-phosphorylated ATM as detected by immunofluorescence localization (Fig. 3a) and by Western blot analysis (Fig. 3b). Moreover, NAC pretreatment (5 mM for 2 h) reduced levels of γH2AX and Ser1981-phosphorylated ATM (Fig. 3a, b). The findings suggest that oxidative DNA damage was likely a predominant factor in the (S)-crizotinib-induced suppression of GC cell growth.

### (S)-crizotinib suppresses gastric cancer cell growth independent of MTH1

**Fig. 4:**
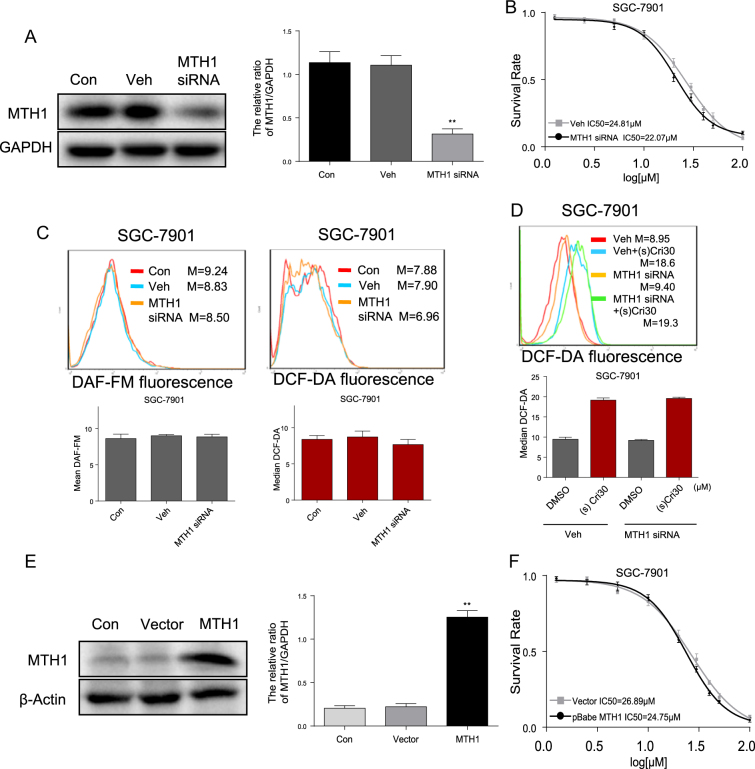
MTH1 knockdown or overexpression does not alter (S)-crizotinib-induced ROS levels or cytotoxicity of gastric cancer cells. **a** SGC-7901 cells were transfected with siRNA target MTH1 sequences; shown is a representative Western blot analysis (left panel); densitometry of the blots (right panel) showing the average ± SEM normalized values *n* = 4, (**p < 0.01). **b** Effects of MTH1 knock-down on on (S)-crizotinib-induced viability was measured by MTT assay; shown are mean absorbance ± SEM, *n* = 4; IC50 values on bottom of graph. **c** Effects of MTH1 knock-down on intracellular levels of ROS as detected by DCFH-DA and nitric oxide as detected by DAF-FM-DA; shown is representative flow cytometric analysis of fluorescent cells (top panel), *m* = mean fluorescence; graph of the mean fluorescence (bottom panel), *n* = 4. **d** Effect of MTH1 knock-down on (S)-crizotinib-induced ROS generation; shown is representative flow cytometric analysis (top panel), m = mean fluorescence; graph of mean fluorescence (bottom panel); *n* = 4. **e** Representative Western blot analysis of MTH1 overexpression in SGC-7901 cells (left panel), β-actin as loading control; denitometric quantification of the blots (right panel) showing mean normalized values ± SEM, *n* = 4 (**p < 0.01). **f** Effects of MTH1 overexpression on (S)-crizotinib-induced cell viability assayed using MTT; shown is mean absorbance ± SEM, *n* = 4, IC_50_ values indicated on bottom of graph

Because evidence indicates that (S)-crizotinib, in contrast to the (R)-enantiomer, is a low nanomolar MTH1 inhibitor^10^, we postulated that MTH1 inhibition is a potential mechanism by which (S)-crizotinib suppresses GC growth. Surprisingly, a 70% siRNA-mediated knock-down of MTH1 protein expression (Fig. 4a) did not prevent the (S)-crizotinib-induced decreased cell viability, with control and siRNA groups yielding similar IC50 values (Fig. 4b). Moreover, MTH1 knock-down alone did not alter intracellular NO (DAF-FM) and ROS (DCF-DA) levels (Fig. 4c) nor affect the ROS level in response to (S)-crizotinib (Fig. 4d). Additionally, a fivefold increase in MTH1 overexpression (Fig. 4e) did not alter the (S)-crizotinib-induced decrease in cell viability (Fig. 4f). The findings indicate that, at least in GC cells, the (S)-crizotinib-mediated ROS activation, suppression of cancer cell growth, promotion of apoptosis, and DNA damage was likely not attributed to MTH1 inhibition.

### (S)-crizotinib activates AKT, a DNA damage response signal

**Fig. 5:**
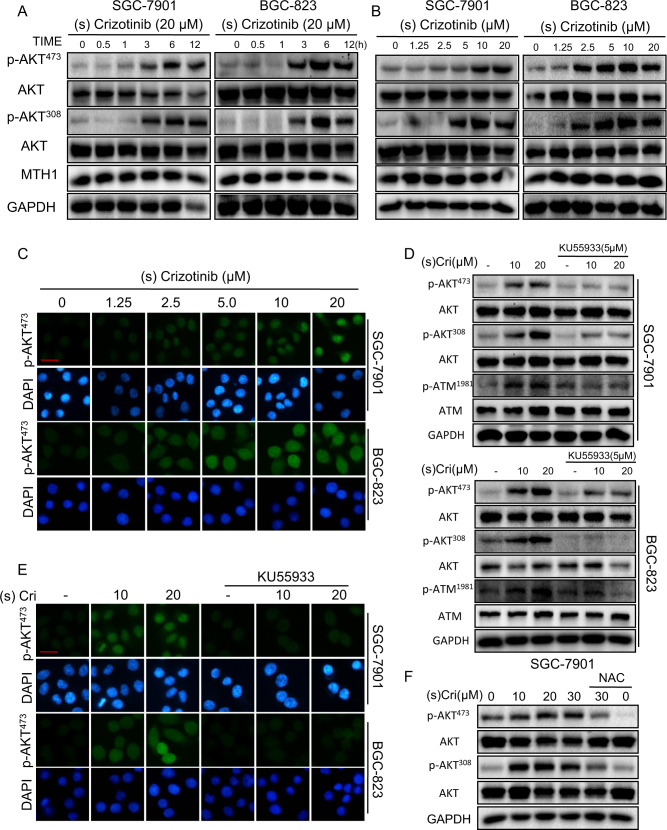
(S)-crizotinib activates Akt, a pro-survival response signal. SGC-7901 and BGC-823 cells were treated with (S)-crizotinib at 20 μM for the indicated times (**a**) or different concentrations for 6 h (**b**). Activation of Akt was determined by Western blot analysis for phosphorylated Akt at Ser473 and Thr308 (pAkt^473^ and pAkt^308^), AKT and GAPDH were used as internal control, detection for MTH1 was also included; *n* = 4. **c** Same experimental groups as in A-B were also used for immunofluorescent detection of activated Akt using p-AKT^473^and p-AKT^308^ antibodies; nuclei were stained with DAPI, *n* = 4 (scale bar = 20 μm). **d** The effect of inhibiting ATM on (S)-crizotinib-induced Akt activation was examined by pretreating cells with 5 µM KU55933 for 2 h before exposing cells to (S)-crizotinib for 6 h, and Western blot analysis (*n* = 4) and **e** immunofluorescent detection for phosphorylated Akt as previously described; *n* = 4 (scale bar = 20 μm). **f** The effects of NAC pretreatment (5 mM for 2 h) on (S)-crizotinib-induced Akt activation were determined by Western blot analysis for. p-AKT^473^ and p-AKT^308^ As previously described, *n* = 4

Our finding that (S)-crizotinib phosphorylated Ser1981 of ATM indicated ATM activation in response to oxidative DNA damage. Activated ATM triggers subsequent survival signals for regulation of cell cycle arrest, repair, apoptosis, and senescence^25^. One of these pro-survival signals is Akt (aka PKB), a key player of cell survival and DNA repair^26,27^. We determined whether (S)-crizotinib activated Akt in the SGC-7901 and BGC-823 cells by determining phosphorylation at Thr308 and Ser473. Western blot analysis revealed that (S)-crizotinib caused time-dependent (Fig. 5a) and dose-dependent (Fig. 5b) increases of phosphorylated Akt at Thr308 and Ser473 (but not (R)-crizotinib (Figure S5A)), which was consistent with increased immunofluorescent localization of phosphorylated Akt (Fig. 5c). Not surprisingly, (S)-crizotinib did not alter MTH1 protein expression levels (Fig. 5a, b) nor did MTH1 knock-down alter Akt expression and phosphorylation (Figure S5B), indicating that these changes induced by (S)-crizotinib are also independent on MTH1.

We next tested the upstream regulation of Akt by ATM using the ATM inhibitor, KU55933. Pretreatment of GC cells with KU55933 (5 μM for 2 h) prevented (S)-crizotinib-induced Akt phosphorylation at Thr308 and Ser473, indicating inhibition of Akt activation (Fig. 5d, e). As positive control, KU55933 inhibited ATM phosphorylation (Fig. 5d). Since NAC suppressed the (S)-crizotinib-induced ATM activation (Fig. 3), it follows that NAC would also inhibit Akt activation. Indeed, pretreatment of GC cells with NAC (5 mM for 2 h) prevented the (S)-crizotinib-induced Akt phosphorylation (Thr308 and Ser473) (Fig. 5f and S6). The findings indicated that (S)-crizotinib, while harboring anti-cancer growth activities, also activated the ATM–Akt pathway for cell survival and DNA repair.

### Akt blockade further enhances anti-cancer activity of (S)-crizotinib

#### Effects of Akt blockade

**Fig. 6:**
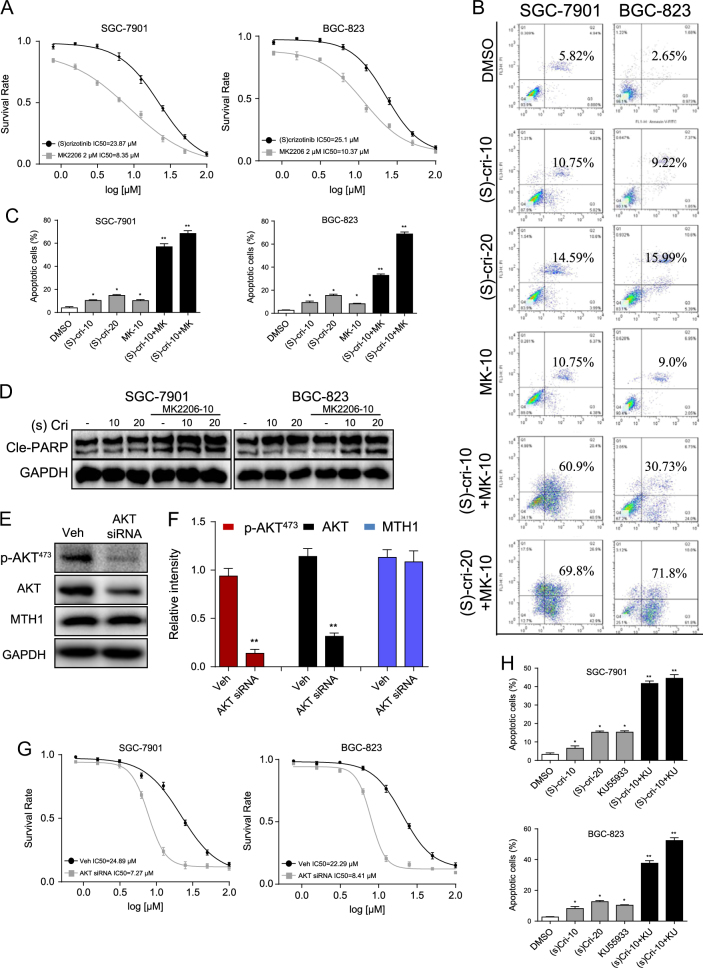
Akt blockade enhances the anti-cancer activity of (S)-crizotinib in gastric cancer cells. The effects of Akt inhibition on the anti-cancer activity of (S)-crizotinib were made by pretreatment of SGC-7901 and BGC-823 cells with MK2206 (2 μM for 2 h), then exposed to increasing concentrations of (S)-crizotinib for 24 h, and cell viability was determined by MTT assay. **a** Shown is the mean ± SEM absorbance units, *n* = 4, IC50 values indicated at bottom of graph. **b** Representative flow cytometric analysis of % apoptotic gastric cancer cells (stained by annexin V/PI) following MK2206 pretreatment, and treated with (S)-crizotinib as indicated; **c** Quantification of apoptotic cells from **b**; shown is average ± SEM, *n* = 4 (*p < 0.05, **p < 0.01). **d** Western blot analysis of cle-PARP in cells from the same treatment groups as **b**; shown is representative blot, GAPDH as loading control; *n* = 4. **e** Gastric cancer cells were transfected with Akt siRNA for 48 h, negative control siRNA (non-targeting) as control (Veh). Western blot analysis for total Akt, phosphorylated Akt at Ser473 (pAkt^473^), and MTH1; GAPDH as loading control. **f** Densitometric quantification of blots in E; shown is average ± SEM of intensity values, *n* = 4 (** *p* < 0.01). **g** Effects of Akt knock-down on (S)-crizotinib-induced cytotoxicity was assessed by the MTT assay; shown is average ± SEM absorbance units, *n* = 4, IC50 values indicated at bottom of graph. **h** Effects of the ATM inhibitor KU55933 on (S)-crizotinib-induced gastric cancer cell apoptosis was assessed by annexin V/PI staining; shown is average ± SEM % apoptotic cells, *n* = 4 (*p < 0.05, **p < 0.01)

Our finding that (S)-crizotinib activated the ATM–Akt pathway suggested that pro-survival signals could, at least in part, masked/countered anti-cancer growth activity exerted on GC cells. We explored this possibility using the allosteric Akt inhibitor, MK2206, which prevents Akt phosphorylation at Thr308 and Ser473, and thereby its activation^28^. The inhibition of Akt phosphorylation with MK2206 in the GC cells was confirmed by Western blot (Figure S7A). MK2206 at 2 µM alone did not affect viability of SGC-7901 and BGC-823 cells (Figure S7B). Results indicated pretreatment with MK2206 (2 µM) further reduced the (S)-crizotinib-induced loss in GC cell viability, lowering the IC_50_ > twofold, and the loss of colony formation (Figure S7C) relative to (S)-crizotinib alone (Fig. 6a). MK2206 pretreatment also enhanced the (S)-crizotinib-induced increased apoptosis by >fourfold (Fig. 6b, c) and enhanced levels of cle-PARP (Fig. 6d and S8) compared to (S)-crizotinib alone.

We further tested the involvement of Akt by its knock-down in the GC cells. Results indicated that a ~70% reduction in expression of non-phosphorylated and phosphorylated Akt (Fig. 6e, f) further lower the (S)-crizotinib-induced reduction of cell viability, lowering the IC50 by ~threefold compared to (S)-crizotinib alone (Fig. 6g). Unsurprisingly, Akt knock-down did not alter MTH1 expression (Fig. 6e, f).

#### Effects of ATM inhibitor

Our finding that the ATM inhibitor, KU55933, prevented the (S)-crizotinib-induced Akt activation suggested that KU55933 also would enhance the (S)-crizotinib-mediated suppression of GC cell growth. Results indicated that KU55933 pretreatment (5 μM for 2 h) of GC cells enhanced the (S)-crizotinib-induced increases in apoptosis by ~threefold compared to (S)-crizotinib alone (Fig. 6h), a finding consistent with increased levels of cell apoptosis (Figure S9A) and cle-PARP (Figure S9B).

#### MK2206 sensitizes a (S)-crizotinib-resistant cell line

**Fig. 7:**
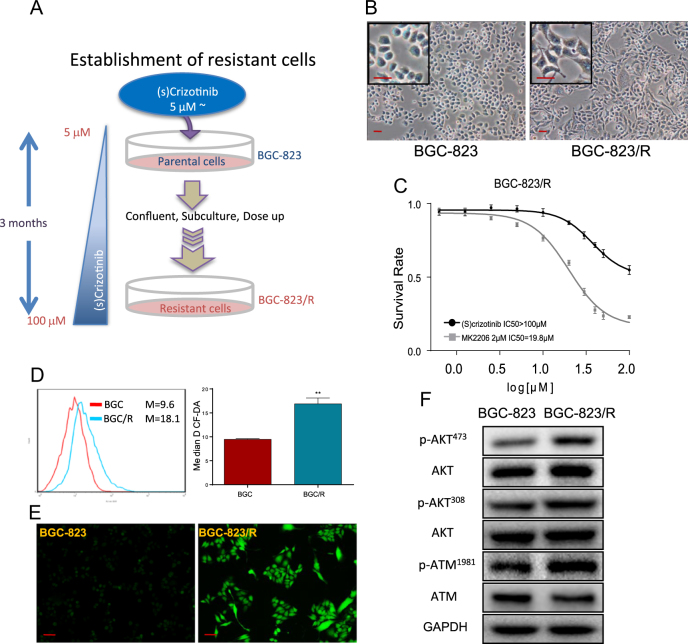
Inhibition of Akt resensitizes resistance cell line to (S)-crizotinib. **a** Schematic showing the generation of (S)-crizotinib-resistant cell line. Parental cells were cultured with stepwise increases in concentrations of (S)-crizotinib, starting with 5 μM, to establish the (S)-crizotinib-resistant line. Each (S)-crizotinib dose was added when normal cell proliferation resumed, thereby continuously enriching the resistant cell pool. Fresh drug was added every 96 h. Resistant cells tolerated 100 μM (S)-crizotinib after 3 months of culture. **b** Morphology of parental and resistant BGC-823 as illustrated by phase contrast microscopy (scale bars = 20 μm). **c** BGC-823/R cells were pretreated with 2 μM MK2206, then exposed to increasing concentrations of (S)-crizotinib for 24 h, and cell viability was assessed by the MTT assay; shown is average ± SEM absorbance units; IC50 values indicated at bottom of graph, *n* = 4. **d** Basal intracellular ROS levels in BGC-823 and resistant BGC-823/R cells were measured by staining with DCFH-DA; representative flow cytometric analysis of DHF fluorescent cells; *n* = 4; **p < 0.01. **e** Basal intracellular ROS in parental and BGC-823/R cells detected by DHF fluorescence and viewed by microscopy (scale bar = 20 μm). **f** Basal levels of p-AKT^473^, p-AKT^308^, and p-ATM^1981^ in BGC-823 and BGC-823/R cells was determined by Western blot analysis. AKT, ATM, and GAPDH were used as the internal controls

Our observation points to a potential role of Akt contributing to development of resistance to (S)-crizotinib and possibly other cancer drugs. We constructed a (S)-crizotinib-resistant cell line BGC-823/R established by long-term culture of BGC-823 cells with (S)-crizotinib (see Materials and methods, Fig. 7a, b). Figure 7c showed that the BGC-823/R cells tolerated high concentrations of (S)-crizotinib, the cell viability with an IC_50_ > 100 µM (versus 25 µM in parental cells). However, the MK2206 pretreatment (2 µM) lowered the IC_50_ to 19.8 µM (Fig. 7c). The BGC-823/R phenotype was characterized by increased basal levels of ROS (Fig. 7d, e) and increased phosphorylated Akt and ATM (Fig. 7f). The findings indicated that Akt inhibition re-sensitized the resistant cells to (S)-crizotinib, suggesting that the pro-survival signals of Akt contributed, at least in part, to the resistance phenotype of the BCG-823/R cells.

### Akt inhibitor further enhances anti-tumor activity of (S)-crizotinib in the xenograft mouse model

**Fig. 8:**
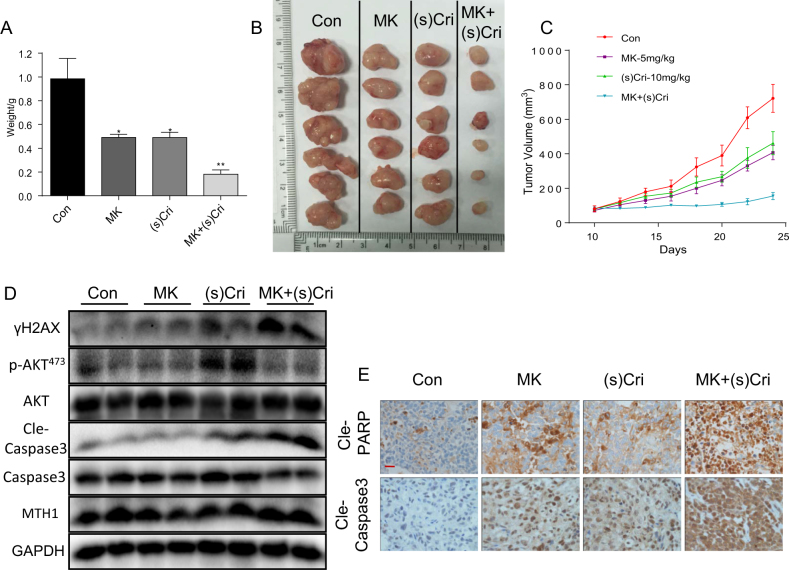
MK2206 enhances the anti-tumor activity of (S)-crizotinib in human gastric cancer xenografts. Gastric tumors were harvested from xenograft mice up to 24 days of treatment with with MK2206 (5 mg/kg, daily), (S)-crizotinib (10 mg/kg, daily), or a combination of both for analysis, *n* = 6 in each group. **a** average ± SEM tumor weight, (*p < 0.05, **p < 0.01), **b** gross images, and **c** average ± SEM tumor volume at indicated time points up to the end of the study. **d** Representative Western blot analysis for detection of γH2AX, phosphorylated Akt at Ser473 (pAkt^473^), Akt, MTH1 and cleaved caspase 3 (cle-caspase 3), caspase 3, and GAPDH as loading control; *n* = 6. **e** Representative immunohistochemical staining of apoptosis markers cleaved-PARP (cle-PARP) and cleaved-caspase 3 (cle-caspase 3); scale bar = 50 μm; *n* = 6

We investigated the anti-tumor effects of (S)-crizotinib or in combination with MK2206 in a human GC xenograft mouse model (see Material and methods). SGC-7901 cells were injected into immunodeficient nude mice, after 10 days, IP injections of (S)-crizotinib (10 mg/kg, daily), MK2206 (5 mg/kg, daily) or combined MK2206 and (S)-crizotinib were made, and tumor growth monitored for 24 days. Examination of tumors indicated that (S)-crizotinib or MK2206 alone reduced the SGC-7901 tumor weight (Fig. 8a), size (Fig. 8b), and volume (Fig. 8c) to similar degrees. However, the combined treatment resulted in significantly greater decreases of the tumor growth indices compare to either treatment alone (Fig. 8a–c). It is interesting that MK2206 alone does not suppress cell growth in vitro but reduces tumor size in vivo. According to our previous study, it should be ascribed to the different concentrations of MK2206 used in vitro and in vivo^29^. Western blot analysis of tumor tissue indicated that (S)-crizotinib increased levels of γH2AX, Akt phosphorylation, and cle-caspase 3, whereas MK2206 alone was without effect (Fig. 8d). The combined treatment enhanced the levels of γH2AX and cle-caspase 3, and, as expected, inhibited Akt phosphorylation (Fig. 8d). The combined treatment also increased the immunohistochemical detection for cle-PARP and cle-caspase 3 (Fig. 8e and S10). The treatment protocol used did not alter the morphology of other organs, i.e., heart, kidney, and liver, nor alter body weight, indicating absence of toxicity in the mice (Figure S11). The finding that Akt blockade enhanced the anti-tumor activity of (S)-crizotinib is consistent with the in vitro data, providing evidence that (S)-crizotinib activated feedback pro-survival signals in the GC cells.

## Discussion

GC is a global disease with high recurrence, poor prognosis, and limited treatment options. We investigated whether (S)-crizotinib, a small-molecule multi-targeted TKI could be a potentially important therapy for the highly heterogeneous disease of GC. The critical findings of this study are that (S)-crizotinib: (a) effectively reduced GC cell viability, promoted apoptosis, induced cell cycle arrest, and decreased xenograft GC tumor growth; (b) inhibited GC cell growth through a mechanism of oxidative DNA damage independent of MTH1 inhibition; and (c) activated the pro-survival Akt–ATM pathway, whose inhibition further enhanced the anti-cancer activity of (S)-crizotinib (Figure S12). The results provide strong support that (S)-crizotinib inhibited GC growth, which are consistent with its anti-cancer activity reported for other cancer cell types^10^. The strong anti-cancer effects of (S)-crizotinib in inhibiting GC cell growth likely indicate that these cells harbor alterations of ALK, ROS1, and or MET, although their precise genomic profile remains to be determined. Additionally, (S)-crizotinib appears to not target non-tumor tissues, a finding consistent with that reported by Huber et al.^10^, an important factor when considering a drug’s side-effects. Furthermore, our findings suggest that inclusion of Akt inhibition (to block pro-survival signaling) with (S)-crizotinib may provide an effective and novel combination therapy for GC in clinical settings with minimal off-target effects.

Our findings indicated that (S)-crizotinib increased generation of ROS and NO in GC cells, implicating an oxidative anti-cancer activity. Crizotinib induces oxdative stress in several cell types, i.e., Rhabdomyosarcoma cell lines^18^, ovarian cancer cells^17^, and cardiomyocytes^19^. The ability of crizotinib, as with other anti-cancer drugs, to produce excessive endogenous levels of oxidants are likely a major factor of its cytotoxicity^30^. In our GC cell model, NAC prevented (S)-crizotinib-induced DNA damage and apoptosis, supporting an anti-cancer mechanism of oxidative DNA damage. The excessive levels of oxidants likely damage DNA by producing single-strand and double-strand breaks, triggering apoptosis^31^. Our finding that (S)-crizotinib-induced phosphorylation of ATM and H2AX, indicating early cellular repair responses to DNA strand breaks^21,32,33^, is consistent with this thesis. Moreover, NAC prevented phosphorylation of ATM, providing further support of an oxidative DNA damage driving the anti-cancer mechanism.

Surprisingly, the oxidative DNA damage in response to (S)-crizotinib was not associated with MTH1 inhibition, despite evidence that (S)-crizotinib is a low nanomolar MTH1 inhibitor^10^. MTH1 belongs to the Nudix hydrolase superfamily, and prevents incorporation of oxidized nucleotides (8-oxo-guanine) into DNA, thereby preventing DNA damage^34^. As such, MTH1is implicated in oncogenic KRAS-driven transformation of lung epithelial cells and evasion of cellular senescence^11^, and its inhibition is thought to promote DNA damage and suppress cancer growth, making MTH1 a potential target for anti-cancer therapies. However, neither MTH1 knock-down nor overexpression in GC cells altered cell viability and intracellular oxidant levels in the absence or presence of (S)-crizotinib, suggesting that MTH1 was not a predominant factor in the anti-cancer mechanism in GC. Our findings are in contrast to those reported by Huber et al.^10^, who observed that MTH1 knock-down increased DNA damage in human colon cancer cells, and overexpression reduced DNA single-strand breaks in response to (S)-crizotinib. The reasons for the contrasting results are unclear, but may be related to differences in cancer cell types and treatment conditions.

The (S)-crizotinib-induced oxidative DNA damage also triggered the pro-survival ATM–Akt pathway, the damage DNA response. Moreover, NAC inhibited activation of ATM and Akt, clearly linking activation to oxidative stress. The blockade of ATM with KU55933 prevented the (S)-crizotinib-induced Akt activation, a finding consistent with reports that ATM is a major regulator of full Akt activation, its inhibition correlated with suppression of Akt-dependent pro-survival signals in cancer cells^25^. Thus, (S)-crizotinib not only promoted anti-cancer activity in GC cells, but also triggered Akt pro-survival signals, leading to inhibition of apoptosis and promotion of cell cycle progression. Significantly, inhibiting the pro-survival ATM–Akt pathway resulted in (i) enhanced anti-cancer activity (S)-crizotinib; (ii) enhanced anti-tumor activity of (S)-crizotinib in the xenograft GC mice, and (iii) re-sensitized resistant cells to (S)-crizotinib. Meanwhile, we found that knocking down Akt does not affect basal cell growth, but treatment with MK2206 alone suppresses basal cell growth. We consider two possible reasons contributing to this interesting phenomenon. (1) Although the expression level of Akt was reduced by siRNA in gastric cancer cells, there was still about 30% of Akt in cells. (2) MK-2206, as a small-molecule Akt inhibitor which binds to the ATP-binding domain of kinase, can also inhibits other kinases (the off-target effects). Above all, the findings strongly suggest that the activated ATM–Akt pathway was countering/masking anti-cancer activity of (S)-crizotinib in the GC.

In summary, we found that (S)-crizotinib inhibited GC cell and tumor growth through a mechanism of oxidative DNA damage independent of MTH1 inhibition. However, (S)-crizotinib also triggered the DNA repair response, activating pro-survival Akt signaling, whose blockade further enhanced anti-cancer activity of (S)-crizotinib in GC and re-sensitized resistant GC cells to (S)-crizotinib. We conclude that inclusion of Akt inhibition with (S)-crizotinib may provide an effective and novel combination therapy for GC in the clinical setting with minimal off-target effects.

## Materials and methods

### Reagents

Akt inhibitor MK2206, ATM inhibitor KU55933, and (S)-crizotinib were obtained from Selleck Chemical (Shanghai, China); (S)-crizotinib was reconstituted in dimethyl sulfoxide (DMSO). *N*-acetyl-l-cysteine (NAC) was purchased from Beyotime Biotech (Nantong, China). Antibodies against cell division cycle protein 2 (Cdc2), cyclin B1, cleaved Poly (ADP-ribose) polymerase (Cle-PARP), murine double minute 2 (MDM-2), GAPDH, and fluorophore- and horseradish peroxidase-conjugated antibodies were purchased from Santa Cruz Biotechnology (Santa Cruz, CA, USA). Antibodies against Akt, phosphorylated Akt (Thr308 and Ser473), ataxia telangiectasia mutated (ATM), Ser1981-phosphorylated ATM, γHA2X, Cleaved-caspase 3 (cle-caspase 3) and MTH1 were purchased from Cell Signaling Technology (Danvers, MA, USA). FITC-Annexin V apoptosis Detection Kit I and Propidium Iodide (PI) were purchased from BD Pharmingen (Franklin Lakes, NJ).

### Cell lines

Human GC cell lines SGC-7901 and BGC-823 were purchased from the Institute of Biochemistry and Cell Biology, Chinese Academy of Sciences. Cells were cultured in RPMI-1640 medium supplemented with 10% heat-inactivated fetal bovine serum, 100 U/mL penicillin and 100 μg/mL streptomycin (Gibco, Eggenstein, Germany).

A (S)-crizotinib-resistant cell line was generated using BGC-823 cells. The cells were continuously cultured with (S)-crizotinib, starting with 5 µM, and increasing stepwise at 96 h intervals to 100 µM to produce enrichment of resistant cells. The cells developed increasing tolerance, and was resistant to (S)-crizotinib at 100 μM by 3 months. Morphologically, BGC-823/R cells displayed cellular projections and phenotype typical of epithelial-to-mesenchymal transition.

### In vitro assays

#### Apoptosis

For apoptosis determination, cells were plated at 2.5 × 10^5^/well in 6-well culture dishes for 24 h and treated with (S)-crizotinib accordingly. Cells were then collected, washed with ice-cold PBS, and evaluated for apoptosis by double-staining with FITC-conjugated Annexin V and PI. Data were collected and analyzed using FACS Calibur flow cytometer.

#### Cell viability

Cells were seeded at a density of 8 × 10^3^/well in 96-well plates, allowed to attach overnight, and treated with (S)-crizotinib at indicated concentrations and times. Following treatment, 0.5 mg/mL of the tetrazolium dye MTT (3-(4,5-dimethylthiazol-2-yl)-2,5-diphenyltetrazolium bromide) was added, incubated for 4 h, and the resultant formation of formazan was dissolved in DMSO. Absorbance was measured at 490 nm using the RT 6000 microplate reader, and %inhibition of was calculated as (1−(treated/ control)) × 100. The IC_50_ values were obtained using the Logit method.

#### Cell cycle analysis

Gastric cancer cells were treated with or without (S)-crizotinib for 15 h, harvested, washed twice with PBS, then fixed in ice-cold 70% (v/v) ethanol for 16 h at 4 °C. Before analysis, cells were washed with PBS, stained with PI according to the manufacturer’s instructions, and incubated for 30 min in darkness at 37 °C. The samples were analyzed by flow cytometry (BD, FACS Calibur, San Jose, CA, USA) using Cell Quest software.

#### Intracellular oxidant detection

Cellular oxidant generation was measured by flow cytometry utilizing fluorescent ROS-sensitive dye, 2’,7’–dichlorofluorescein diacetate (DCFH-DA) and nitric oxide (NO)-sensitive dye, 4-amino-5-methylamino-2’,7’-DAF-DA (Beyotime Biotech, Nantong, China). Briefly, 5 × 10^5^/plate were plated in 6-well culture dishes, cultured overnight in normal growth medium, and treated with (S)-crizotinib at concentrations and times indicated. Following treatment, cells were incubated with 10 μM DCFH-DA for detection of ROS or 5 μM DAF-FM-DA for detection of NO at 37 °C for 30 min, harvested and washed with PBS, and fluorescence was measured by flow cytometry (FACS Calibur, BD Biosciences, CA). In some experiments, cells were pretreated with 5 mM NAC for 2 h. In all experiments, 8000 viable cells were analyzed. For some experiments, ROS generation was detected from cells plated on glass slides, and treated with DCFH-DA for cellular ROS detection, and images viewed and recorded by fluorescence microscopy.

#### Colony formation assay

Cells were seeded at 500 cells/well in 6-well plates, treated with MK-2206 (10 µM), (S)-crizotinib (at 2.5 or 5 µM), or combined and cultured for 8 days. The cells were stained with crystal violet (0.5 in 25% methanol) to assess colony growth. A colony is defined as a cluster of at least 50 cells determined microscopically.

### siRNA knock-down and overexpression

The siRNA duplexes used in this study were purchased from Invitrogen (Carlsbad, CA, USA). Target siRNAs were custom designed based on the following sequences:

MTH1 (5’-CGACGACAGCUACUGGUUU-3’) and Akt (5’-GCACUUUCGGCAAGGUGAUdTdT-3’). Negative Universal Control (Invitrogen, Carlsbad, CA) was used as the control. Cells were seeded at 3 × 10^5^/well into 6-well plates, cultured for 24 h, and transfected with target or control siRNAs (100 nM final concentration) using lipofectamine 2000 (Invitrogen, Carlsbad, CA). Forty-eight hours after transfection, cells were collected, and degree of knock-down was determined by detected by Western blot analysis of MTH1 and Akt proteins.

For overexpression, cells were transfected with MTH1 cDNA (the forward primer 5’-AGTGTGGTGGAATTCATGAGTGGAATTAGCCCTCA-3’ and the reverse primer 5’-ATATCTGCAGAATTCGGACCGTGTCCACCTCGCGG-3’) using an expression vector with an EcoRI restriction site (Invitrogen). Forty-eight hours following transfection, lysates were prepared and the level of MTH1 expression determined by Western blot analysis.

### Western blot analysis

Cells or tumor tissues were homogenized in protein lysis buffer and protein concentrations determined by the Bradford assay (Bio-Rad, Hercules, CA). Protein samples were separated using 6–12% sodium dodecyl sulfate-polyacrylamide gels, transferred to PVDF membranes, and blocked with 5% nonfat milk for 1 h at room temperature. Blots were probed with specific primary antibodies, detected with horseradish peroxidase-conjugated secondary antibodies, and visualized using ECL kit (Bio-Rad, Hercules, CA). The density of the immunoreactive bands was analyzed using Image J software (NIH, Bethesda, MD).

### Immunofluorescence cell staining

Cells plated on glass slides were fixed with 4% paraformaldehyde, permeabilized with 0.1% Triton X-100, and incubated with primary antibodies at 1:200 dilution overnight at 4 °C. Fluorophore-conjugated secondary antibody (1:200) was used for detection, and DAPI as nuclear stain.

### Immunohistochemistry

Tumor tissues were fixed in 10% formalin at room temperature, embedded in paraffin, and sectioned at 5 μm thickness. Tissue sections were rehydrated, incubated with primary antibodies at 4 °C overnight, followed by conjugated secondary antibodies and diaminobenzidine (DAB) for detection. Images were captured using Image-Pro Plus 6.0 (Media Cybernetics, Inc, Bethesda, MD). Liver, kidney, and heart tissues from mice were stained with hematoxylin and eosin (H&E).

### In vivo xenograft model

Protocols for the mouse studies were approved by the Wenzhou Medical College Animal Policy and Welfare Committee and followed the National Institutes of Health guide for the care and use of Laboratory animals. Five-week-old, athymic BALB/c nu/nu female mice (17–19 g) were purchased from Vital River Laboratories (Beijing, China), housed at constant room temperature with a 12 h:12 h light/dark cycle and fed a standard rodent diet with water ad libitum. SGC-7901 cells were injected subcutaneously into the right flank of mice with 1 × 10^7^ cells in 150 μL of PBS. At day 10 post injection, mice developed tumors reaching 50–100 mm^3^ volume, and intraperitoneal (IP) injections made as follows: (1) control vehicle, (2) Akt inhibitor MK2206, 5 mg/kg daily, (3) (S)-crizotinib once daily (10 mg/kg), and (4) combined MK2206 and (S)-crizotinib (6 mice in each group). At time points up to 24 days, mice were sacrificed, tumors harvested and weighed. Samples were prepared for histology and Western blot analysis. Tumor volumes were determined by measuring length (*l*) and width (*w*) to calculate volume (*V* = 0.5 × *l* × *w*^2^).

### Statistics

The studies were randomized and blinded, and data reported as mean ± SEM from at least three independent experiments. Statistical analysis was performed with GraphPad Prism 5.0 software (San Diego, CA, USA), using one-way ANOVA followed by Dunnett’s post-hoc test when comparing more than two groups of data. We applied one-way ANOVA, non-parametric Kruskal–Wallis test followed by Dunn’s post-hoc test when comparing multiple independent groups. When comparing two groups, the unpaired *t*-test was used. Statistical significance is defined at *P* value < 0.05.

### Data availability

All other data are included within the Article or Supplementary Information or available from the authors on request.

## Electronic supplementary material

Supplementary file
