# Supplementary material for: (S)-crizotinib reduces gastric cancer growth through oxidative DNA damage and triggers pro-survival akt signal
Source: Cell Death Dis. 2018 May 31;9(6):660. doi: 10.1038/s41419-018-0667-x (PMC5981313; doi:10.1038/s41419-018-0667-x)
Supplement: Supplementary file 1 — Supplementary file [file 41419_2018_667_MOESM1_ESM.docx]

***Supporting Information***

**(S)-crizotinib reduces gastric cancer growth through oxidative DNA damage and triggers pro-survival Akt signal**

Jiansong Ji^1,2,#^, Weiqian Chen^1,2,#^, Weishuai Lian^3^, Ruijie Chen^4^, Jinqing Yang^5^, Qianqian Zhang^1,2^, Qiaoyou Weng^1,2^, Zia Khan^1^, Jie Hu^1^, Xi Chen^1^, Peng Zou^1^, Xiaoming Chen^5,*^, Guang Liang^1,*^

^1^ Chemical Biology Research Center, School of Pharmaceutical Sciences, Wenzhou Medical University, Wenzhou, Zhejiang 325035, China

^2^ Department of Interventional Radiology, The Fifth Affiliated Hospital of Wenzhou Medical University, Lishui, Zhejiang 323000, China

^3^ Department of Interventional and Vascular Surgery, Shanghai Tenth People’s Hospital, Tongji University, Shanghai 200072, China

^4^ Department of Pharmacy, The Second Affiliated Hospital of Wenzhou Medical University, Wenzhou, Zhejiang 325000, China

^5^ The First Affiliated Hospital of Wenzhou Medical University, Wenzhou, Zhejiang 325035, China

**SUPPLEMENTARY DATA**

**Figure S1. Effects of** **(S)-crizotinib on the viability and morphology of human gastric cancer cells.**

Phase contrast image of gastric cancer cells (SGC-7901 or BGC-823) treated with (S)-crizotinib at 10, 20, or 30 µM, respectively, (s)Cri-10, (s)Cri-20 or (s)Cri-30; Con=control. Sparsely distributed cells with rounded morphology can be seen in SGC-7901 and BGC-823 cells following exposure to (S)-crizotinib for 24 hr [scale bar = 20 μm]. Representative data were shown from three independent experiments.


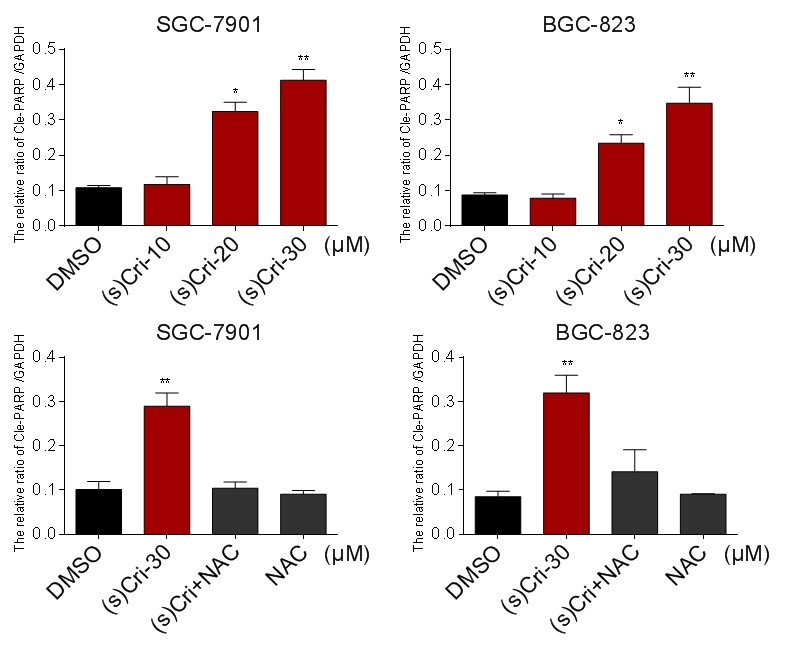


**Figure S2.** Densitometric quantification for Figure 1D (n=3 in each group, * p < 0.05, ** p < 0.01).

**Figure S3.** **(S)-crizotinib induces ROS generation in human gastric cancer cells.**

SGC-7901 and BGC-823 cells were pretreated with 5 mM NAC for 2 hr before exposure to (S)-crizotinib at 10, 20 or 30 μM for 30 min [respectively, (s)Cri-10, (s)Cri-20, (s)Cri-30]. Intracellular ROS generation was detected by dichlorodihydrofluorescein fluorescence. [green = dichlorodihydrofluorescein; scale bar = 20 μm]. Representative data were shown from three independent experiments.


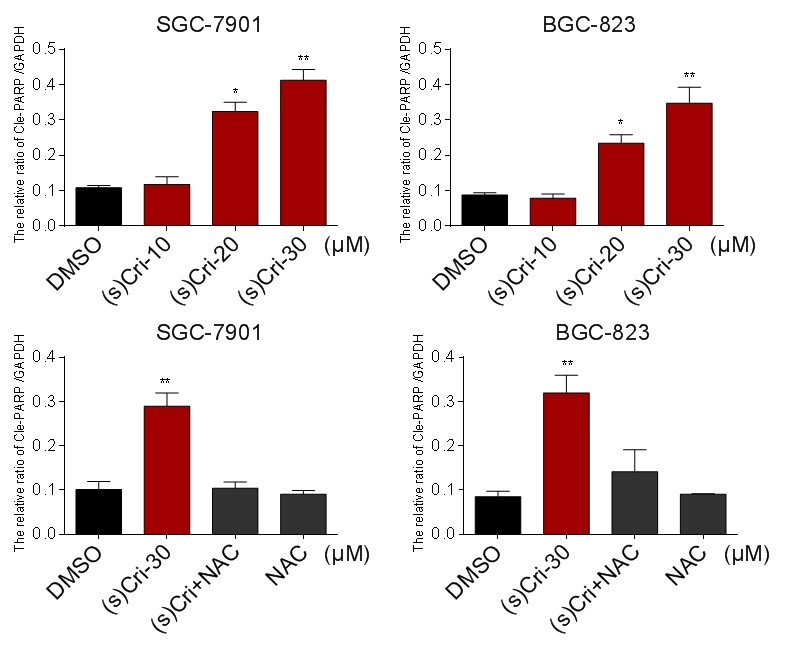


**Figure S4.** Densitometric quantification for Figure 2G (n=3 in each group, * p < 0.05, ** p < 0.01).


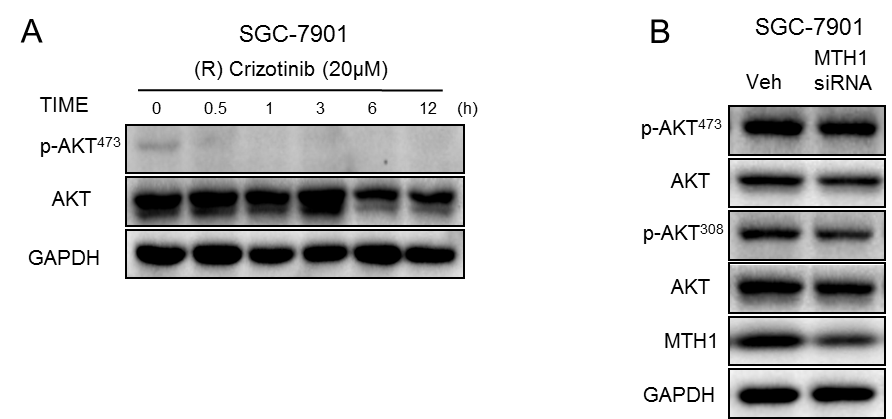


**Figure S5. Akt phosphorylation is not mediated by (R)-crizotinib.** (**A**) SGC-7901 cells were treated with (R)-crizotinib at 20 μM for the indicated times. Western blot analysis for p-AKT^473^ was determined. GAPDH and AKT were used as the internal controls. (**B**) SGC-7901 cells were transfected with siRNA against MTH1 or non-targeting control siRNA (Veh) for 48 hr, and Western blot analysis for p-AKT^473^, p-AKT^308^ and MTH1 were determined. Representative data were shown from three independent experiments.


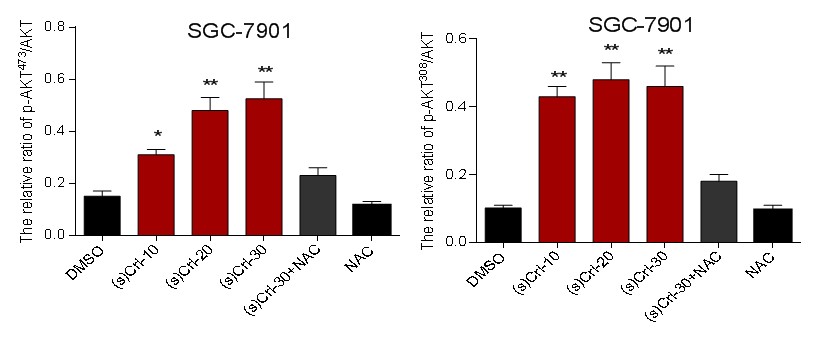


**Figure S6.** Densitometric quantification for Figure 5F (n=3 in each group, * p < 0.05, ** p < 0.01).


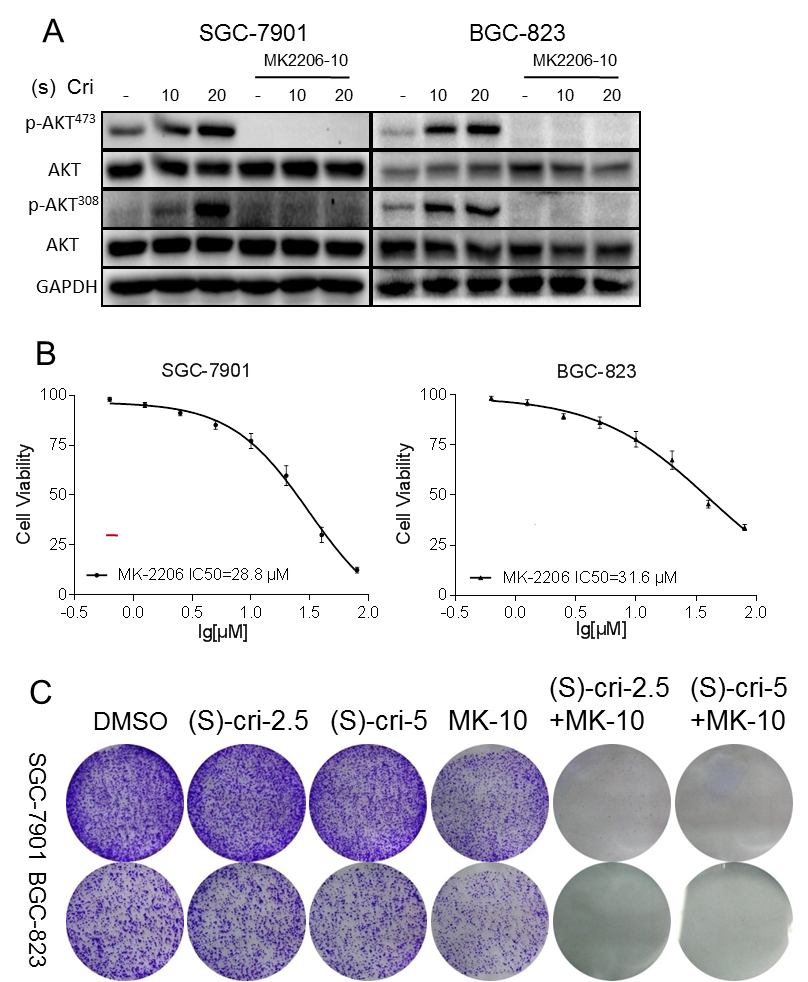


**Figure S7. Akt inhibition enhances the anti-tumor activity of** **(S)-crizotinib in gastric cancer cell lines.** (**A**) The effect of Akt inhibitor, MK2206, on basal and (S)-crizotinib-induced Akt phosphorylation in SGC-7901 and BGC-823 cells. Cells were pretreated with 10 μM MK2206 for 2 hr, followed by increasing doses of (S)-crizotinib for 6 hr, and collected for Western blot analysis. Representative Western blot analysis for p-AKT^473^,p-AKT^308^, and unphosphoryalated Akt determined; GAPDH as loading control, n=4. (**B**) The effect of Akt inhibition by MK2206 on gastric cancer cell viability was assessed by the MTT assay. Gastric cancer cell lines SGC-7901 and BGC-823 were treated with increasing concentrations of MK2206 (0.625-80 μM) for 24 hr; shown is average+SEM absorbance value, IC50 values indicated in bottom of graph. (**C**) Effect of (S)-crizotinib and MK2206 combined treatment on gastric cancer cell colony formation. Cells were stained with crystal violet after 14 days of treatment. Representative data were shown from three independent experiments.


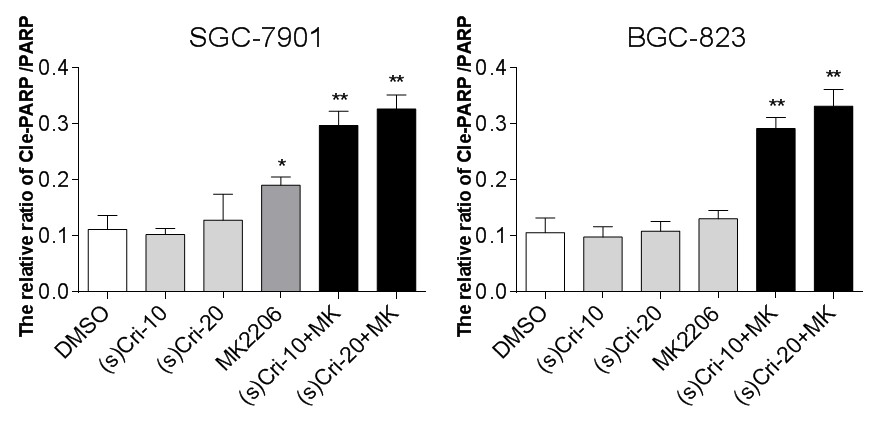


**Figure S8.** Densitometric quantification for Figure 6D (n=3 in each group, * p < 0.05, ** p < 0.01).


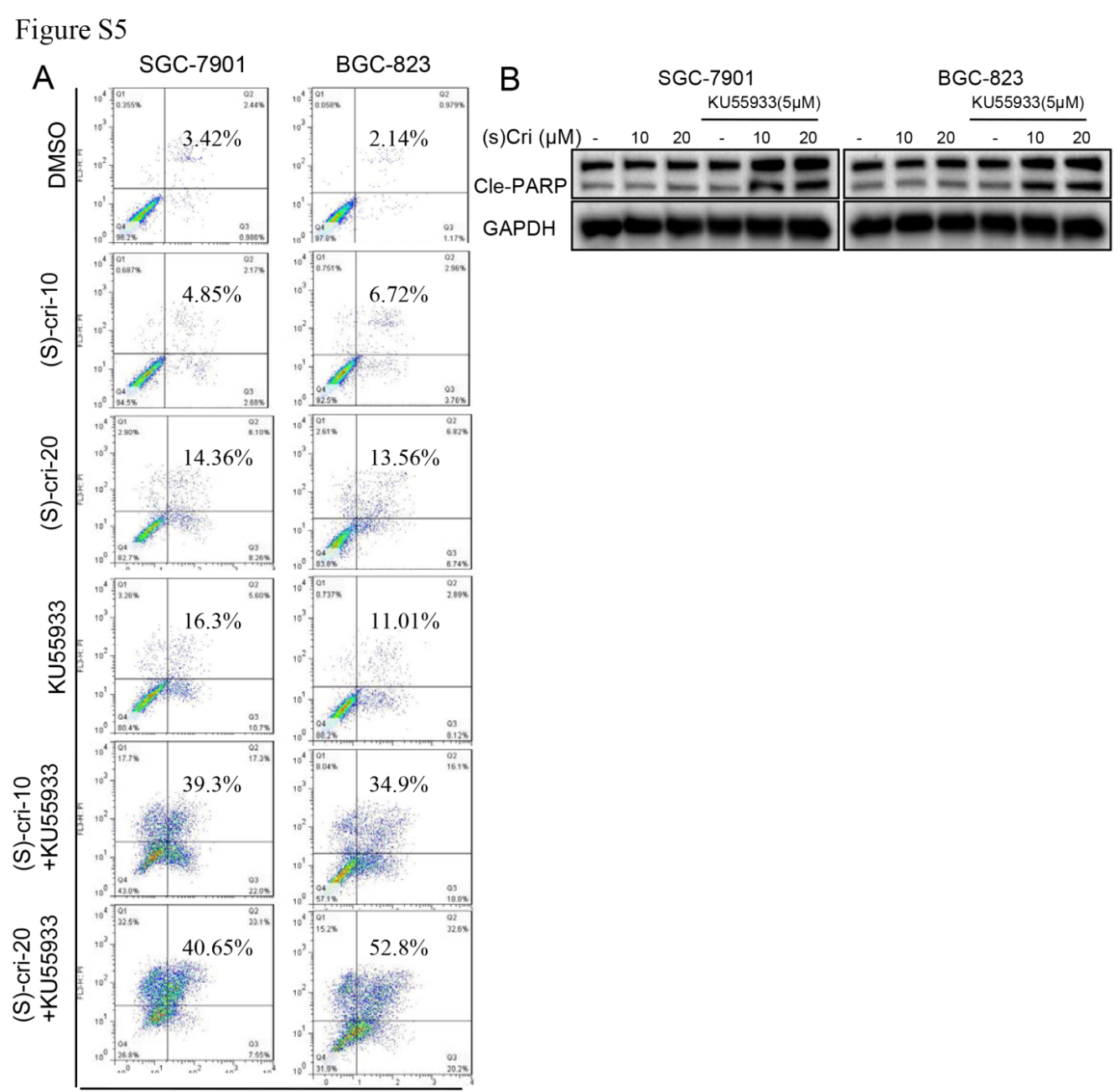


**Figure S9. ATM inhibitor KU55933 enhances (S)-crizotinib-induced apoptosis in human gastric cancer cells.** (**A**) SGC-7901 and BGC-823 cells were pretreated with KU55933 (5 μM for 2 hr), followed by (S)-crizotinib treatment with 10 or 20 µM [(s)Cri-10, (s)Cri-20, respectively], or with (s)-crizotinib or KU55933 alone, and apoptosis assessed by annexin V/PI staining; shown is representative flow cytometric analysis of % apoptotic cells; n=4. (**B**) Effects of KU55933 on (S)-crizotinib-induced cleaved-PARP (cle-PARP) levels were evaluated by Western blot analysis; shown is representative blot of cleaved-PARP, with GAPDH as loading control; n = 3.


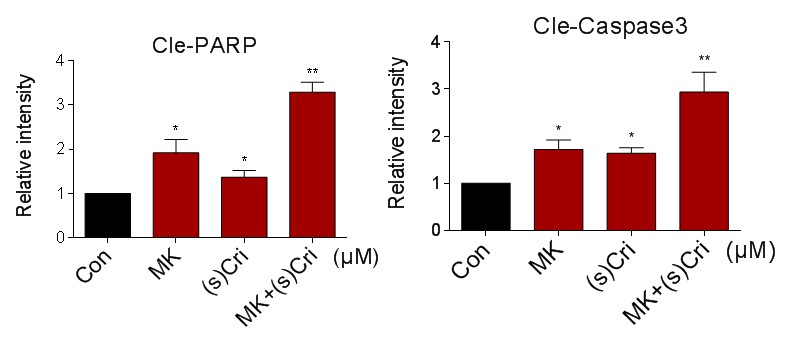


**Figure S10.** Quantification for staining results in Figure 8E and represented as the percent of control (n=6 in each group, * p < 0.05, ** p < 0.01).


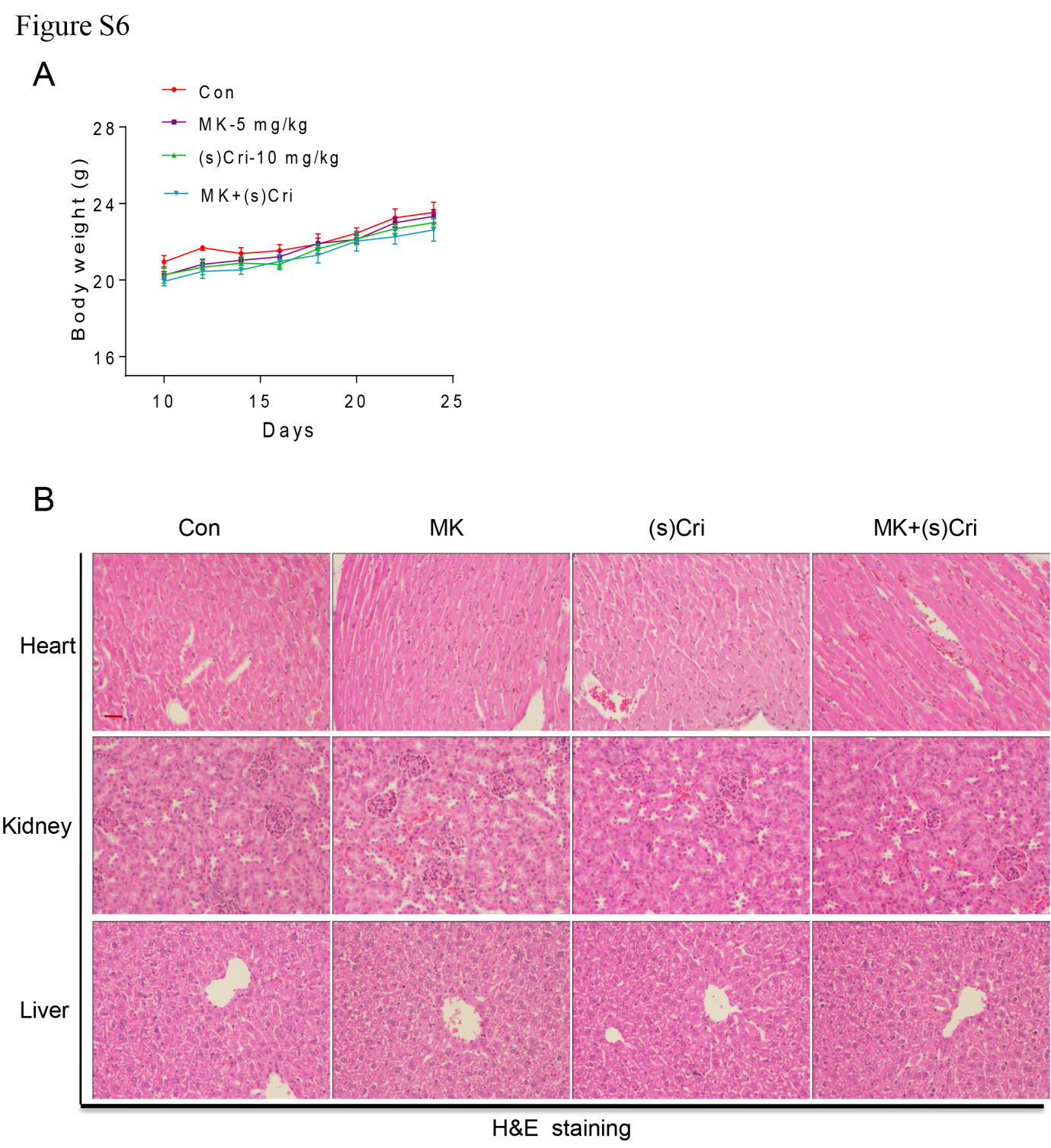


**Figure S11. Absence of toxicity in non-tumor tissues of xenograft mice treated with (S)-crizotinib and MK2206.** (**A**) Body weight of xenograft mice treated with (S)-crizotinib [(s)-Cri] or MK2206 (MK) alone or in combination for 24 days; shown is average + SEM gram (g) body weight. (**B**) Heart, kidney, and liver tissues from the treatment groups of the xenograft mice in A were stained with hematoxylin and eosin (H&E) [n=6]. Con=control; [scale bar = 50 μm].


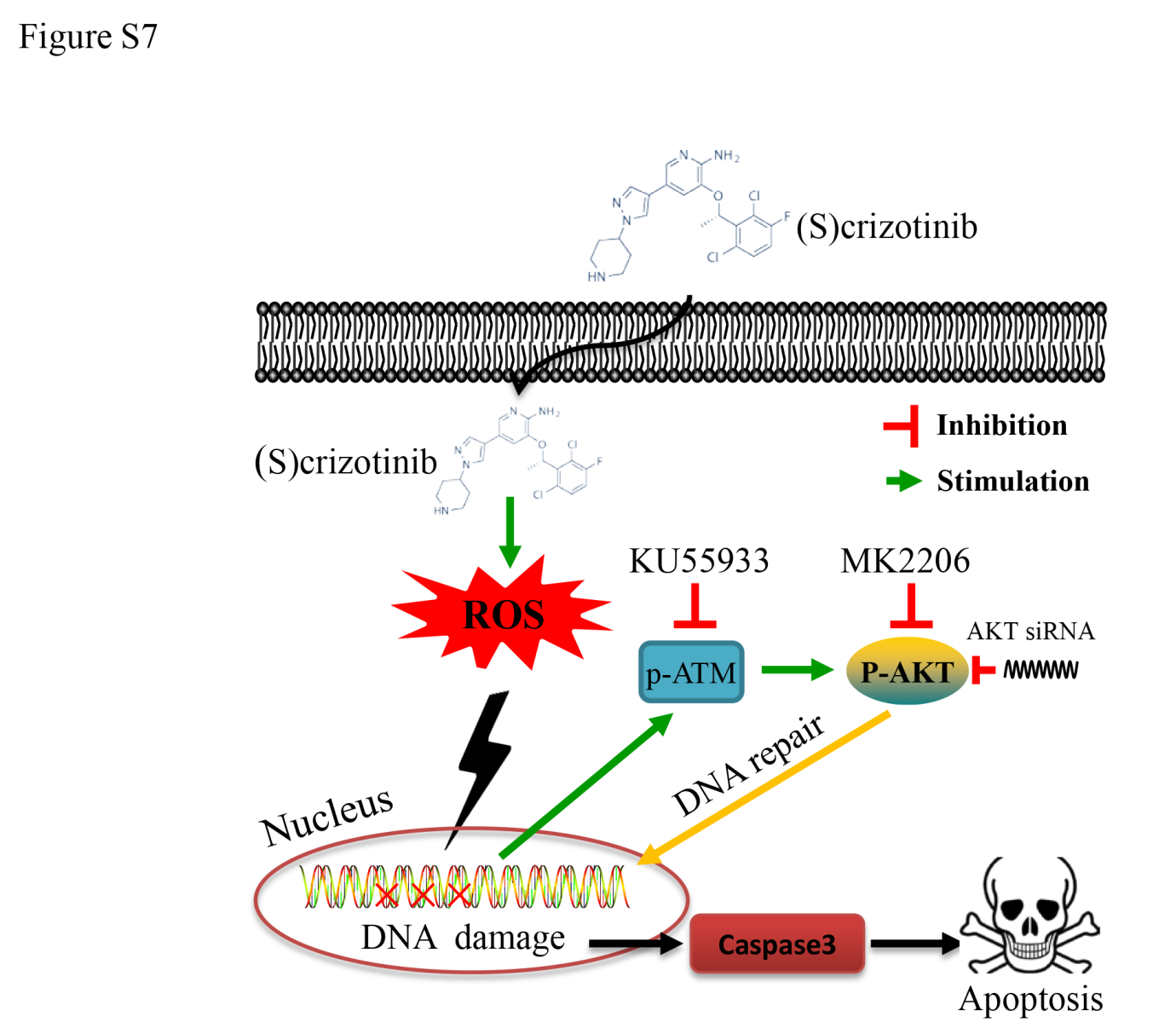


**Figure S12. Schematic illustration of the major findings.** (S)-crizotinib induces reactive oxygen species (ROS), leading to oxidative DNA damage and apoptotic cell death. DNA damage triggers the DNA repair response, one early response is activation of the serine/threonine kinase, ataxia telangiectasia mutated (ATM). The autophosphorylated ATM in turn, activation phosphorylates and activates protein kinase B/Akt protein, a key pro-survival signal can inhibit apoptosis, promote cell cycle progression, and DNA repair. Hyperactivation of Akt may be the driving force in (S)-crizotinib resistance, a thesis further supported by our observation that BGC-823/R cells have higher p-AKT^473^ and p-AKT^308^ than parental cells. Our working model suggests that inclusion of Akt inhibition with (S)-crizotinib treatment may provide an effective and novel strategy in gastric cancer therapy.
